# EP4 and Class III β-Tubulin Expression in Uterine Smooth Muscle Tumors: Implications for Prognosis and Treatment

**DOI:** 10.3390/cancers11101590

**Published:** 2019-10-18

**Authors:** Jocelyn Reader, Amy K. Harper, Teklu Legesse, Paul N. Staats, Olga Goloubeva, Gautam G. Rao, Amy Fulton, Dana M. Roque

**Affiliations:** 1 Division of Gynecologic Oncology, University of Maryland School of Medicine, Baltimore, MD 21201, USA; jreader@som.umaryland.edu; 2 University of Maryland Marlene and Stewart Greenebaum Comprehensive Cancer Center, Baltimore, MD 21201, USA; grao@som.umaryland.edu (G.G.R.); afulton@som.umaryland.edu (A.F.); 3 Department of Obstetrics, Gynecology, and Reproductive Sciences, University of Maryland School of Medicine, Baltimore, MD 21201, USA; aharper4@med.wayne.edu; 4 Department of Pathology, University of Maryland School of Medicine, Baltimore, MD 21201, USA; Teklu.Legesse@som.umaryland.edu (T.L.); pstaats@umm.edu (P.N.S.); 5 Department of Epidemiology and Public Health, University of Maryland School of Medicine, Baltimore, MD 21201, USA; OGoloubeva@som.umaryland.edu; 6 Baltimore Veterans Affairs Medical Center, Baltimore, MD 21201, USA

**Keywords:** class III β tubulin, PGE2, EP4, leiomyosarcoma, uterine, tumors

## Abstract

The microtubule-stabilizing agent docetaxel in combination with gemcitabine represents one of the most effective regimens against the aggressive gynecologic tumor leiomyosarcoma (LMS). Upregulation of class III β-tubulin has previously been shown to confer taxane resistance in a variety of human cancers. Prostaglandin E_2_ receptor EP4 is linked to progression of a variety of human cancers and may represent a novel target for tumor inhibition in LMS. We evaluated the hypotheses that EP4 and class III β-tubulin have increased expression in LMS in comparison to normal myometrium or benign tumors and that expression of class III β-tubulin correlates with resistance to taxanes and poor clinical outcome. Gene expression was examined using TCGA data and correlated with clinicopathologic outcome which demonstrated that class III β-tubulin is more highly expressed in more aggressive sarcomas with EP4 being widely expressed in all subtypes of sarcoma. Immunohistochemistry for EP4 and class III β-tubulin was performed on patients with LMS, leiomyomatosis/STUMP, leiomyoma, and normal myometrium. Expression of EP4 and class III β-tubulin were characterized for cell lines SK-UT-1, SK-UT-1B, and PHM-41 and these cell lines were treated with docetaxel alone and in combination with EP4 inhibitors. In taxane-resistant cell lines that overexpress class III β-tubulin and EP4, treatment with EP4 inhibitor resulted in at least 2-fold sensitization to docetaxel. Expression of class III β-tubulin and EP4 in LMS may identify patients at risk of resistance to standard chemotherapies and candidates for augmentation of therapy through EP4 inhibition.

## 1. Introduction

In 2019, approximately 61,880 women in the United States will be diagnosed with a malignancy of the uterine corpus. Approximately 12,160 die from their disease annually [1]. Gynecologic sarcomas represent a minority (8%) of these cases but carry a disproportionately poor prognosis across all stages. Leiomyosarcoma (LMS) is the most common uterine sarcoma, representing 40–60% of cases [2]. Development of effective novel therapeutic approaches requires a more thorough understanding of sarcoma tumor biology.

Microtubules, dynamic cytoskeletal structures with many important roles within the cell including separation of chromosomes during mitosis, transport, locomotion, and maintenance of cell structure, are a major target of chemotherapeutic agents [3]. The microtubule component β-tubulin is potentially expressed as nine isoforms. In benign tissues, expression of the constitutive form (class I) is ubiquitous, whereas class III β-tubulin is generally restricted to normal neural tissues [4]. Upregulation of class III β-tubulin confers taxane resistance in colon, prostate, breast, and ovarian/uterine carcinosarcoma and serous carcinoma, among others [5,6,7,8,9], in part due to alterations in the binding pocket conferring reduced affinity of paclitaxel and docetaxel [10]. Overexpression of class III β-tubulin in vulvar LMS cell line, SK-LMS-1, leads to resistance to another microtubule targeted chemotherapeutic eribulin [11]. Overexpression of class III β-tubulin has robustly been associated with chemoresistance and poor clinical outcome.

LMS is among the most aggressive gynecologic sarcomas, with 5-year disease-specific survival ranging from 76% (stage I) to <29% (stage IV) [12]. For locally advanced disease, surgical resection followed by chemotherapy is the preferred treatment for LMS. Gemcitabine plus docetaxel currently represents one of the most effective cytotoxic combinations for this disease, with an overall response rate (ORR) of 27% [13]. Ifosfamide (ORR 17%) [14], pegylated liposomal doxorubicin (ORR 16%) [15], paclitaxel (ORR 8%) [16], oral etoposide (ORR 7%) [17], cisplatin (ORR 5%) [18], and ixabepilone (0% ORR) [19] have also been evaluated in patients. Adjuvant radiation therapy does not result in improved survival [20]. The prognosis for LMS remains dismal, and the need for novel treatment strategies is imperative.

**Figure 1:**
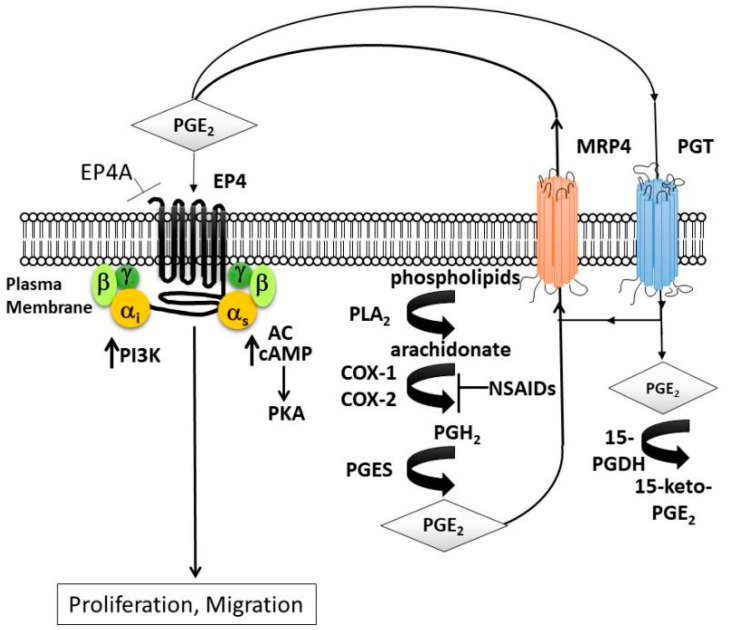
Prostaglandin signaling. Phospholipase A2 (PLA_2_) converts phospholipids from the plasma membrane to arachidonate. Cyclooxygenase enzymes, (COX-1 or COX-2) catalyze arachidonate to prostaglandin H2 (PGH_2_) followed by conversion to prostaglandin E2 (PGE_2_) by PGE synthase. PGE_2_ is extracellularly exported via the multidrug-resistance-associated protein-4 (MRP4) where it binds to four G-protein-coupled receptor subtypes (EP1–EP4), coupled to different intracellular signaling pathways. EP4/EP2 are linked to cyclic AMP (cAMP) and protein kinase A (PKA) via G_αs_ and adenylate cyclase (AC). EP4 also activates phosphoinositide-3-kinase (PI3K) through G_αi_. PGE_2_ is imported inside the cell via prostaglandin transporter (PGT) and converted to an inactive form 15-keto-PGE_2_ by 15-hydroxyprostaglandin dehydrogenase (15-PGDH). The use of an EP4 antagonist (EP4A) could act to reduce proliferation and migration.

The cyclooxygenase pathway (COX) (Figure 1) provides an opportunity for targeted tumor inhibition. COX exists as two isoenzymes: constitutive COX-1 and inducible COX-2. Prostaglandin E2 (PGE_2_) is the principle product of the COX enzymes and may be upregulated in inflammation or tumorigenesis. PGE_2_ can interact with one of four G protein coupled receptors: EP1, EP2, EP3, or EP4. By binding with EP4 specifically on the malignant cell, PGE_2_ can induce the PKI3/AKT and cAMP/PKA pathways, inhibit apoptosis, and promote proliferation, angiogenesis, cell migration, lymphangiogenesis and stem-cell like functions [21,22,23]. Pharmacologic targeting of the EP4 receptor can halt this cascade. In preclinical models, EP4 inhibitors have shown promise for several cancers including breast and prostate [24,25].

Activation of the COX pathway may play a role in sarcoma biology. In Kaposi’s sarcoma, signaling initiated by PGE_2_ through EP receptors may trigger malignant transformation [26]. In osteosarcoma, a recent meta-analysis found that COX-2 expression predicted reduced 2-year overall and disease-free survival, without significant association with age, gender, tumor location, histology, stage, metastasis, or necrosis [27]. COX-2 has been shown to be upregulated in uterine carcinosarcoma [28].

To our knowledge, there is no information regarding expression of either class III β-tubulin or EP4 in uterine smooth muscle tumors. In this study, we sought to characterize protein expression of class III β-tubulin and EP4 across a spectrum of gynecologic stromal tumors (LMS, smooth muscle tumor of uncertain malignant potential (STUMP), disseminated leiomyomatosis, leiomyoma) and to seek correlations with subsequent chemoresponsiveness to docetaxel or EP4 inhibition as a single agent or in combination. In this exploratory study, we tested the hypotheses that (1) sarcomas overexpress EP4 relative to normal myometrium, and tumors with aggressive behavior (i.e., LMS) express higher levels of class III β-tubulin and EP4 than those with indeterminate (i.e., STUMP, leiomyomatosis) or benign (i.e., leiomyoma, normal myometrium) behavior; thus, EP4 inhibition may represent a novel targeted therapy for LMS; and (2) expression of class III β-tubulin correlates with resistance to taxanes and poor clinical outcome.

## 2. Results

**Figure 2:**
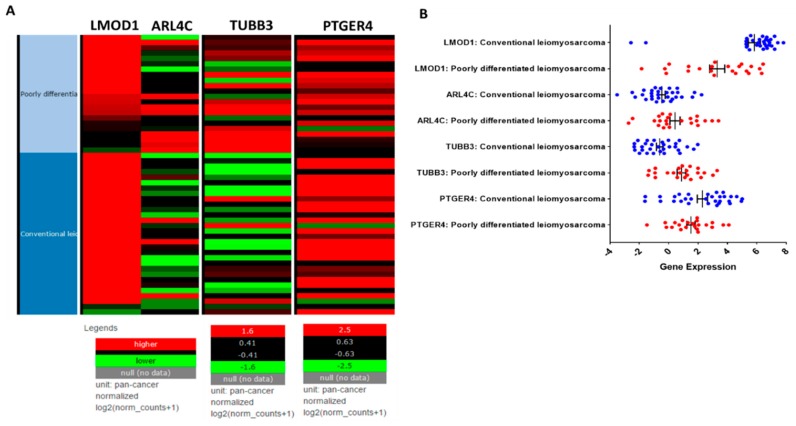
Gene expression analysis of leiomyosarcoma (LMS) from the TCGA. (**A**) Gene expression heat map for *LMOD1, ARL4C, TUBB3,* and *PTGER4* from conventional (dark blue) or poorly differentiated (light blue) leiomyosarcoma obtained from the cancer genome atlas (TCGA). Red indicates higher gene expression and green lower gene expression. Samples were restricted to female patients with LMS from gynecologic and retroperitoneal areas (*n* = 54). *LMOD1* and *ARL4C* are biomarkers for conventional and poorly differentiated subtypes, respectively. (**B**) Mean gene expression for *LMOD1, ARL4C, TUBB3,* and *PTGER4* segregated by LMS subtype. Gene expression for conventional LMS indicated in blue and for poorly differentiated LMS in red. Heat map image was modified from the Xena Browser [31].

The role of *TUBB3* and *PTGER4* has not been investigated in uterine LMS. While LMS is relatively rare, it has a very poor prognosis; therefore, we decided to analyze the gene expression of *TUBB3* and *PTGER4* in 54 primary LMS tumor samples from the TCGA representing conventional and poorly differentiated LMS (Figure 2). The genes *LMOD1* (leiomodin-1) and *ARL4C* (ADP ribosylation factor-like 4C) have previously been identified as markers for conventional (good outcome) (Figure 2A, dark blue) and poorly differentiated (poor outcome) (Figure 2A, light blue) LMS, thus we investigated possible relationships between *LMOD1* and *ARL4C* to *TUBB3* and *PTGER4* [29,30]. In the TCGA cohort, *LMOD1* was more highly expressed, indicated as red in the heat map (Figure 2A), in conventional LMS than in poorly differentiated LMS with a mean gene expression of 5.9 vs. 3.3, respectively (Figure 2B). *ARL4C* was more highly expressed in poorly differentiated LMS than in conventional LMS with a mean gene expression 0.4 vs. −0.6, respectively (Figure 2B). The differences in gene expression of *LMOD1* and *ARL4C* in conventional and poorly differentiated LMS confirms previously published data [29,30] (Figure 2).

Analogous to *ARL4C*, *TUBB3* was also more highly expressed in poorly differentiated LMS compared to conventional LMS (mean gene expression 0.9 vs. −0.6) (Figure 2B). *PTGER4* had increased expression in both subtypes of LMS with gene expression values of 2.2 for conventional and 1.5 for poorly differentiated LMS. Multivariable analysis of variance with all four biomarkers in the general linear model between conventional and poorly differentiated subtypes revealed that the subtypes differ in regard to the marker distribution with an overall test statistic of F_1,52_ = 16.67 and *p* = 0.0002. Pairwise comparison indicates that *LMOD1* (*p* = 0.0002), *TUBB3* (*p* = 0.0001), and *ARL4C* (*p* = 0.01) have different to very different gene expression levels between conventional and poorly differentiated LMS; in contrast, *PTGER4* (*p* = 0.14) is expressed in both subtypes of tumors with no significant difference between the two subtypes (Table 1). These data support the notion that *TUBB3* expression correlates with poor clinical prognosis and that *PTGER4* is commonly expressed in LMS. This study also demonstrates, for the first time, that *PTGER4* expression is increased in both subtypes of LMS (Figure 2).

### 2.1. LMS Expresses Class III β-Tubulin and EP4

Given the results from the TCGA analysis, we identified a total of 29 cases of uterine smooth muscle tumors from our institution in order to analyze protein expression of class III β-tubulin and EP4. Twelve normal myometrium cases served as controls. Patient and disease characteristics are provided in Table 2. Our patient population is similar to the results of a recent analysis of 13,089 cases based on the SEER database which showed a higher incidence of LMS in black compared to white women [32]. The normal myometrial control group was 83% white and 17% black. Nine of 10 LMS cases presented as stage 3 or 4 disease, which was higher than the reported average of 45% [32].

**Figure 3:**
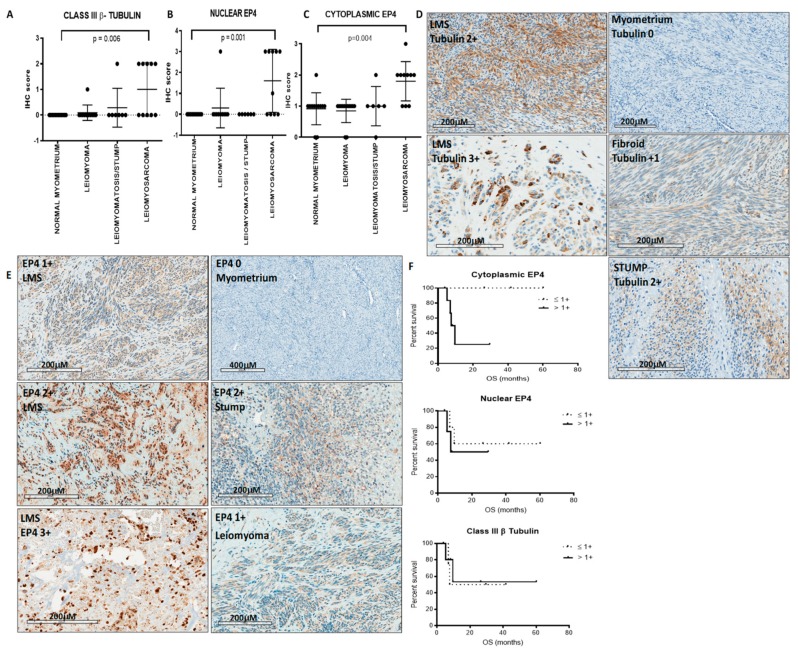
Protein expression in solid tissues. (**A**) Leiomyosarcoma overexpressed class III β-tubulin relative to samples of normal myometrium (*p* = 0.006), leiomyoma, and leiomyomatosis/STUMP. (**B**) Immunohistochemistry scores for cytoplasmic (*p* = 0.001) and (**C**) nuclear (*p* = 0.004) EP4 expression were higher in LMS compared to normal myometrium and other smooth muscle tumors. (**D**,**E**) Representative immunohistochemistry staining for tubulin and EP4 in normal myometrium and uterine stromal tumors. (**F**) Survival analysis of >1+ cytoplasmic EP4 expression suggests a trend for poorer overall survival (top). Differences in overall survival were not observed in nuclear EP4 expression (middle) or in class III β-tubulin expression (bottom); however due to insufficient events from the small number of cases statistical analysis of survival cannot be performed.

Class III β-tubulin immunoreactivity was scored in the cytoplasmic compartment and both cytoplasmic and nuclear compartments were evaluated for EP4 immunoreactivity. Immunoreactivity was graded on a scale of 0 (no staining) to 3+ (strong). Class III β-tubulin is highly expressed in LMS compared to normal myometrium (*p* = 0.006) and leiomyoma (n.s.) in which no tubulin was detected (Figure 3A). Additionally, class III β-tubulin was highly expressed in LMS compared to leiomyomatosis/STUMP in which 50% of LMS has 2+ expression vs. only 12.5% leiomyomatosis/STUMP exhibiting 2+ expression (Figure 3A). Immunohistochemistry scores for EP4 expression were also higher in LMS (median 2+) compared to smooth muscle tumors and normal myometrium (median 1+) (Figure 3B,C). For both nuclear (*p* = 0.001) and cytoplasmic (*p* = 0.004) subcellular locations, EP4 is more highly expressed in LMS compared to normal myometrium. Representative immunohistochemistry staining is shown in Figure 3D,E. Cytoplasmic EP4 expression (>1+) was associated with poorer overall survival (Figure 3F). Neither nuclear EP4 nor class III β-tubulin expression correlated with survival differences.

### 2.2. Carcinosarcoma Cell Lines SK-UT-1 and SK-UT-1B Overexpresses Class III β-Tubulin and EP4 in Comparison to Normal Myometrium

**Figure 4:**
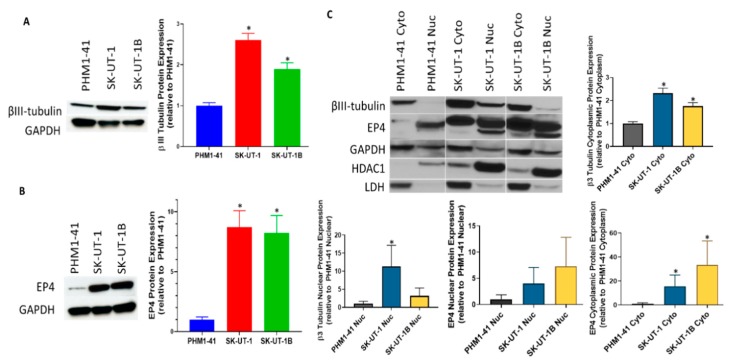
Protein expression in cell lines. (**A**) Class III β-tubulin and (**B**) EP4 protein expression were analyzed via Western blot in sarcoma (SK-UT-1) and carcinoma (SK-UT-1B) cell lines relative to normal immortalized uterine myometrial cell line PHM1-41. Both class III β-tubulin and EP4 were found to be overexpressed compared to PHM1-41. The bar graphs on the left indicate densitometry measurement of the Western blots. Protein expression analysis of the nuclear and cytoplasmic fractions is shown in (**C**). The bar graphs below on the right indicate densitometric measurement of cytoplasmic and nuclear class III β-tubulin or EP4 proteins normalized to GAPDH. Both class III β-tubulin and EP4 proteins in the cytoplasmic fractions of SK-UT-1 and SK-UT-1B had significantly more expression compared to PHM1-41. Only class III β-tubulin in the nuclear fraction of SK-UT-1 had significantly increased expression compared to PHM1-41. The following antibodies were used as loading controls: GAPDH (total protein), histone deacetylase 1 (HDAC1) (nuclear loading control), and lactate dehydrogenase (LDH) (cytoplasmic loading control). * *p* < 0.01.

Based on the data demonstrating increased expression of the EP4 protein in LMS compared to normal myometrium and leiomyoma and on the account that EP4 has not been previously explored in LMS, we evaluated the total protein expression of EP4 (Figure 4B) and class III β-tubulin (Figure 4A) in uterine sarcoma (SK-UT-1), uterine carcinoma (SK-UT-1B), and immortalized normal myometrium (PHM1-41) cell lines. SK-UT-1 and SK-UT-1B cell lines represent the mesenchymal and epithelial components from a uterine carcinosarcoma derived from a single patient. Class III β-tubulin was previously evaluated in vulvar LMS cell line SK-LMS-1 but not in uterine derived LMS cell lines. As measured by densitometry, EP4 was significantly overexpressed in SK-UT-1 and SK-UT-1B by approximately 9- and 8-fold higher, respectively, compared to PHM1-41 cells (Figure 4B). Likewise, SK-UT-1 and SK-UT-1B significantly expressed approximately 2.5- and 2-fold more class III β-tubulin, respectively, relative to PMH1-41 (Figure 4A). Expression of both proteins were higher in the sarcoma cell line vs. the carcinoma cell line which is consistent with a more aggressive clinical phenotype.

Based on the presence of both cytoplasmic and nuclear immunoreactivity of EP4 in patient uterine tumor samples, class III β-tubulin and EP4 protein expression within the cytoplasmic and nuclear fractions of PHM1-41, SK-UT-1, and SK-UT-1B were also evaluated (Figure 4C). The majority of the EP4 protein is detected in the nuclear fraction of PHM1-41, whereas expression is seen in both the cytoplasmic and the nuclear fractions of malignant cell lines. Interestingly, cytoplasmic EP4 expression was associated with poorer overall survival, and while EP4 was found mostly in the nuclear fraction with very little cytoplasmic expression in the normal PHM1-41 cell line, the malignant LMS cell lines both demonstrated increased expression of EP4 in both the cytoplasmic and nuclear fractions, thus exhibiting similar expression patterns to what was observed in primary LMS tissue (Figure 3E and Figure 4C). Class III β-tubulin was readily identified within the cytoplasmic, and, to a lesser degree, within the nuclear fractions of uterine sarcoma (SK-UT-1) and carcinoma (SK-UT-1B) cell lines; and in normal myometrium (PHM1-41), expression was largely limited to the cytoplasmic fraction (Figure 4C). Similarly, expression for both class III β-tubulin and EP4 was significantly increased in the cytoplasmic portions of SK-UT-1 and SK-UT-1B, replicating what was observed for the total protein Western blots. Class III β-tubulin also had a significant increase in expression in the nuclear fraction of SK-UT-1. There was also a noticeable trend for higher expression in the nuclear fractions for EP4 expression. In primary LMS tumors, class III β-tubulin was observed in the cytoplasmic compartment but was not reported in the nuclear compartment. While there have not been reports of nuclear class III β-tubulin expression, class II β-tubulin has been shown to be present in the nucleus of non- and less-differentiated normal human epidermal keratinocyte cells [33]. We have shown that LMS cell lines have increased expression of both EP4 and class III β-tubulin compared to normal myometrium and the expression and location of these proteins reflects what is observed in primary LMS tumor tissue.

### 2.3. EP4 Antagonists Inhibit Migration of SK-UT-1 and SK-UT-1B

**Figure 5:**
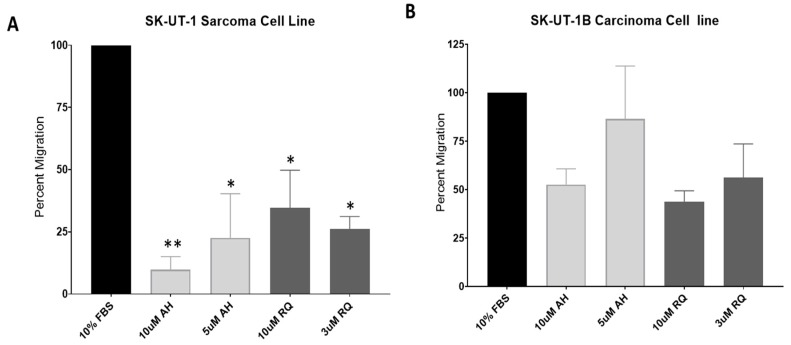
Effect of EP4 antagonists AH23848 and RQ15986 on SK-UT-1 and SK-UT-1B migration. (**A**,**B**) Addition of EP4 antagonists AH23848 and RQ15986 lead to a decrease in migration in SK-UT-1 and SK-UT-1B. Data are normalized to the FBS control (100%) and presented in percent migration. *AH: AH23848. RQ: RQ15986.* Statistics: two-way ANOVA with Dunnett’s test. * *p* < 0.01, ** *p* < 0.001.

EP4 is known to promote the migration and metastasis of many malignant cells [25]. We investigated the effects of EP4 inhibition on the migration of SK-UT-1 and SK-UT-1B cell lines using two different EP4 antagonists. SK-UT-1 cells exhibited a decrease in migration in a dose responsive manner by nearly 90% in the presence of the EP4 antagonist AH23848 (10 µM, *p* < 0.01; 5 µM, *p* < 0.05). The EP4 antagonist RQ15986 also lead to a decrease in the migration of SK-UT-1 by 75%; however, for unknown reasons, we did not observe inhibition in a dose responsive manner (10 µM and 3 µM, *p* < 0.05) (Figure 5A). For SK-UT-1B, migration was decreased by up to 50% in a dose-dependent response with either AH23848 or RQ15986; however, we did not reach statistical significance compared to the positive control (Figure 5B). As a mono-therapy, treatment of SK-UT-1 and SK-UT-1B leads to a decrease in cellular migration.

### 2.4. Pre-Treatment with EP4 Antagonists Enhances Sensitivity to Docetaxel

**Figure 6:**
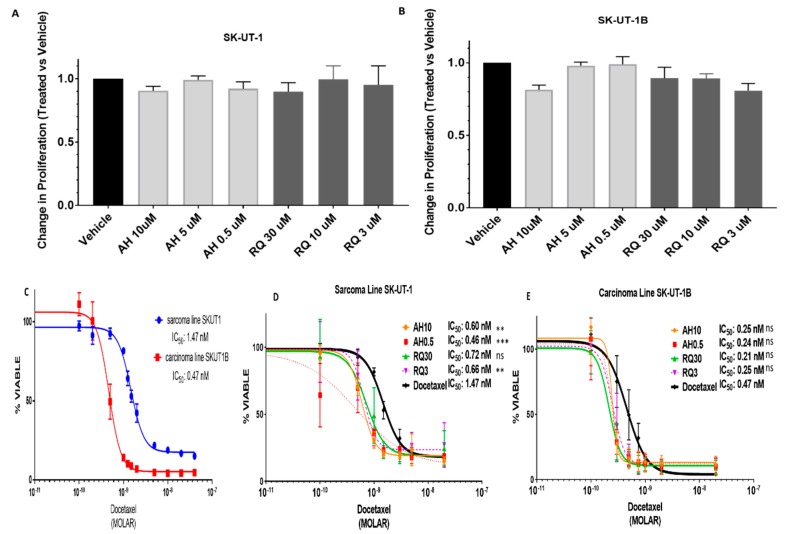
Effect of EP4 antagonists AH23848 and RQ15986 on SK-UT-1 and SK-UT-1B on proliferation and response to docetaxel (**A**,**B**) There was a modest effect on cell proliferation on SK-UT-1 (7% decrease, n.s.) and SK-UT-1B (20% decrease, n.s.). Data are normalized to the vehicle control. Error bars represent experiments performed in triplicate. (**C**) Sarcoma cell line SK-UT-1 (IC_50_ 1.5 nM) was more chemoresistant to docetaxel than carcinoma cell line SK-UT-1B (IC_50_ 0.5 nM). (**D**,**E**) Pre-treatment with EP4 antagonist (either AH23848 or RQ15986) increased chemosensitivity to docetaxel in both cell lines by approximately 2-fold. Statistics: one-way ANOVA with post hoc analysis. Combination drug treatment compared to docetaxel. *AH: AH23848. RQ: RQ15986.* ** *p* < 0.01, *** *p* < 0.001.

We also assessed if EP4 antagonists would have any effect on proliferation. Using a DNA content-based assay, we observed negligible effects on proliferation in SK-UT-1 and SK-UT-1B when treated with AH23848 or RQ15986 (Figure 6A,B). The modest effect on proliferation has been observed in other cells lines treated with EP4 antagonists including breast and bladder cancer [25,34]. First-line treatment for gynecologic LMS is often a combination of docetaxel with gemcitabine; thus, we tested the effect of EP4 antagonists in combination with docetaxel on SK-UT-1 and SK-UT-1B. IC_50_ values for docetaxel as a mono-therapy in SK-UT-1 and SK-UT1B were approximately 1.5 nM and 0.5 nM, respectively (Figure 6C). Pre-treatment with EP4 antagonists AH23848 or RQ15986 resulted in a significant increase in sensitivity to treatment with docetaxel in SK-UT-1 cells (Figure 6D). A 2-fold sensitization occurred when cells were treated with AH23848 with a decrease in the IC_50_ from 1.47 nM for docetaxel as a single agent to 0.6 nM (*p* < 0.01) when combined with 10 µM AH23848 and 0.46 nM (*p* < 0.001) when combined with 0.5 µM AH23848. We observed similar trends with another EP4 antagonist, RQ15986, when SK-UT-1 was treated in combination with docetaxel in which the IC_50_ decreased to 0.72 nM (N.S.) when treated with 30 µM RQ15986 and 0.66 nM (*p* < 0.01) when treated with 3 µM RQ15986 compared to an IC_50_ of 1.47 nM for single agent docetaxel (Figure 6D). A similar, albeit not a statistically significant, effect was observed in SK-UT-1B cells. The difference in chemosensitization between SK-UT-1 and SK-UT-1B when pre-treated with EP4 antagonists may be due to differences in EP4 protein expression since SK-UT-1 has higher EP4 expression compared to SK-UT-1B (Figure 6E). We also did not observe a dose-dependent response with the EP4 antagonists due to unknown reasons. These data show that combination therapy with EP4 antagonist and docetaxel is superior to docetaxel single-agent treatment in LMS cell lines.

## 3. Materials and Methods

### 3.1. Patients

This study was approved by the Institutional Review Board (HP-0061673; HP-0063458). Cases of leiomyosarcoma, STUMP, leiomyomatosis, leiomyoma, and normal myometrium were identified retrospectively from 2009–2015 within a single institution. Patient’s clinical and demographic characteristics were obtained. Progression-free survival (PFS) was defined as the time from diagnosis to the earlier of disease recurrence or death; overall survival (OS) was defined as time from diagnosis to date of death from any course censored at date of last contact. Patients were not used for the experiments thus patient consent forms were not required. All procedures were performed in accordance with the ethical standards of the institution and/or national research committee and with the 1964 Helsinki declaration and its later amendments or comparable ethical standards.

### 3.2. Immunohistochemistry

Immunohistochemistry staining was performed after antigen-retrieval using either rabbit anti-PTGER4/EP4 (LS-A3898, LifeSpan BioSciences, Seattle, WA, USA) or Class III β-tubulin (TUJ1, BioLegend, San Diego, CA, USA). Primary antibody was omitted in the negative control. Immunoreactivity was graded as 0 (no staining), 1+ (weak), 2+ (moderate), or 3+ (strong), and the percentage of tumor cells stained (focal: 0–25%, intermediate: 25–75%, or diffuse: >75%). Immunoreactivity was scored for the cytoplasmic compartment for class III β-tubulin, and for cytoplasmic and nuclear compartments for EP4. Staining was scored by two pathologists (P.N.S., T.L.) blinded to clinical outcomes.

### 3.3. Cell Lines

Cell lines were obtained from ATCC (Manassas, VA, USA) and maintained at 37 °C degrees, 5% CO_2_. The biological characteristics of a sarcoma cell line (SK-UT-1) were analyzed in comparison to well-differentiated adenocarcinoma (SK-UT-1B) and uterine myometrial smooth muscle (PHM1-41) cell lines. SK-UT-1 and SK-UT-1B represent the malignant mesenchymal and epithelial components from a uterine grade III mesodermal mixed tumor consistent with leiomyosarcoma (carcinosarcoma) derived from a 75-year-old, female, Caucasian patient.

### 3.4. Western Blot Analysis

Total protein lysates were generated using RIPA buffer (Sigma, St. Louis, MO, USA) according to manufacturer’s instructions, supplemented with protease inhibitors and separated on 10% TGX gel (Bio-Rad, Hercules, CA, USA) under reducing conditions. The gel was transferred to a PVDF membrane and probed with 1:1000 EP4 antibody (Cayman Chemical, Ann Arbor, MI, USA), 1:1000 anti-tubulin β-3 (TUJ1; BioLegend) or 1:10,000 GAPDH (CST, Danvers, MA, USA). Densitometry was analyzed with ImageJ: https://imagej.nih.gov/ij/. Subcellular fractions were generated using the NE-PER Nuclear and Cytoplasmic Extraction kit according to manufacturer’s instructions (ThermoScientific, Waltham, MA, USA). Experiment was performed in triplicate and representative images are shown.

### 3.5. Drugs

Two EP4 antagonists were used AH23848 (Cayman Chemical) and RQ15986 (AskAt, Inc., Japan). Docetaxel was purchased from Cell Signaling Technology (CST, Danvers, MA, USA).

### 3.6. Proliferation Assays

Cells (PHM1-41: 2000 cells/well; SK-UT-1: 2000 cells/well, SK-UT-1B: 4000 cells/well) were seeded on black well, clear bottom 96-well plates (Greiner Bio-One North America Inc., Monroe, NC, USA) and treated with EP4 antagonists for 72 h. Proliferation was assessed using CyQuant Direct Cell Proliferation Assay (ThermoFisher Scientific, Pittsburgh, PA, USA) according to manufacturer’s instructions. For dual treatment studies, cells were seeded in various concentrations of EP4 antagonist, allowed to attach, and treated with docetaxel 24 h later. Each condition was performed in triplicate and each experiment was performed at least three times.

### 3.7. Migration Assays

SK-UT-1 and SK-UT-1B cells were serum-starved and pre-treated with vehicle or EP4 antagonists for 4 h. Twenty-five thousand cells were seeded per well in a Cultrex cell migration assay (Trevigen, Gaithersburg, MD, USA) with fetal bovine serum (positive control), serum-free media (negative control) or media containing various concentrations of EP4 antagonists. Migration was analyzed 12 h later. Doubling times for SK-UT-1 and SK-UT-1B have been reported to be between 72–96 h which is well beyond the 12-h migration assay [35]. Each condition was tested in triplicate per experiment and the migration experiment was repeated three times.

### 3.8. Bioinformatics Analysis

Gene expression data from The Cancer Genome Atlas (TCGA) sarcoma cohort was downloaded for analysis of the *TUBB3*, *PTGER4*, *LMOD1,* and *ARL4C* genes using the UCSC Xena platform [31]. Cases were restricted to females with LMS ‘gynecologic’ or ‘retroperitoneal’ locations. The multivariable analysis of variance approach was utilized (general linear model, GLM) to test for plausible differences in gene expression between conventional and poorly differentiated LMS subtypes. Testing was done at the 0.05 level of significance.

### 3.9. Statistical Analyses

Dose–response curves were analyzed, and IC_50_ was defined as the concentration of drug required to result in reduction of cell viability to 50% of vehicle control. IC_50_ was determined through interpolation of sigmoidal curves fit with a standard Hill slope of −1.0. The Kaplan–Meier approach was utilized to estimate time-to-event parameters. Overall survival (OS) was defined as time form diagnosis to death from any cause censored at date of last contact. Progression-free survival (PFS) was defined from time of diagnosis to the first recurrence or death without recurrence. The estimated time-to-event functions were compared using the log rank test. Nonparametric Kruskal–Wallis approach was applied to estimate and compare the distributions of the median of the immunohistochemistry scores followed by Dunn’s multiple comparisons test to compare the IHC score of normal myometrium to each group. Protein expression data were analyzed using Student’s *t*-test. Migration data were analyzed by the two-way ANOVA implementing the Dunn’s multiple comparisons procedure. Statistical tests were conducted at the 0.05 level of significance. Analyses were done using the GraphPad Prism 7.0 (GraphPad Software, La Jolla, CA, USA), and SAS (v. 9.4, Institute Inc., Cary, NC, USA).

## 4. Discussion

Smooth muscle tumors are the most common mesenchymal tumors in the female genital tract. Uterine smooth muscle tumors have a broad spectrum ranging from LMS to STUMP to leiomyomas and these tumors can present with similar symptoms and are classified by histopathologic criteria [36,37,38]. Representing one end of the spectrum are uterine leiomyomas, benign estrogen-receptor positive tumors which affect 70–80% of all women [39]. Less commonly, leiomyomas may also be found multifocally in extrauterine locations (i.e., leiomyomatosis); the pathogenetic mechanisms underlying this phenomenon have yet to be described [40]. Leiomyomas typically have low cellularity, minimal cellular atypia, and mitoses <5/5 high power field (hpf); exceptions to each of these criteria constitute variants such as highly cellular/mitotically active leiomyomas or leiomyoma with bizarre nuclei. Necrosis is not seen. On the opposite end of the spectrum, LMS is defined by the presence of at least two of three features: coagulative necrosis, mitoses >10/10 hpf, and/or significant nuclear atypia. In between these extremes, STUMP tumors, with recurrence rates approaching 17%, exhibit intermediate behavior, but do not fulfill criteria for definitive diagnosis of LMS [41]. Uterine sarcomas can present as either pure sarcoma or a mixed sarcoma and carcinoma and historically there have been few systemic treatment options for this rare disease [38,42].

In this study, we characterized the expression of class III β-tubulin and EP4 across a spectrum of uterine smooth muscle tumors in order to examine markers of chemoresistance and to establish the mechanistic plausibility of EP4 antagonism as a novel therapeutic strategy. Currently, the most active chemotherapeutic combination against LMS consists of a taxane (docetaxel) in combination with gemcitabine, a nucleoside analog of deoxycytidine resulting in chain termination [13,43,44]. Nevertheless, response rates to this combination are no better than 27%, and complete responses in the setting of recurrent disease are rarely reported. The mechanisms underlying chemoresistance in LMS are not well elucidated. Overexpression of class III β-tubulin has been linked to taxane resistance in a variety of other primary cancers. Recently, Yahiro and colleagues hypothesized a role for class III β-tubulin in eribulin-resistant vaginal LMS [11]. Compared to parental SK-LMS-1, the eribulin-resistant cell line overexpressed class III β-tubulin (*TUBB3*) and demonstrated cross resistance to other microtubule-interacting drugs such as vinca alkaloids or taxanes. However, knockdown of *TUBB3* only partially recovered sensitivity to eribulin which suggests that class III β-tubulin is not solely responsible for the observed eribulin resistance in SK-LMS-1. In our study, we chose to focus on uterine tumors; therefore, we utilized a uterine myometrial smooth muscle cell line, PHM1-41, along with two cell lines originating from the same tumor from a single patient representing a mixed uterine sarcoma/carcinoma, SK-UT1 and SK-UT-1B. We also observed increased expression of class III β-tubulin in both the sarcoma and carcinoma cell lines when compared to normal myometrium cell line PHM1-41, with the sarcoma cell line expressing higher levels, a 2.5-fold increase, compared to the 2 fold increase observed in the carcinoma cell line. We determined that the sarcoma cell line SK-UT-1 was also more resistant to docetaxel exhibiting an IC_50_ of 1.47 nm compared to 0.47 nm exhibited by SK-UT-1B.

LMS frequently exhibits necrosis, with the potential to incite an inflammatory cascade, which suggests a role for the COX pathway to influence biology of this tumor. Though pre-clinical data have suggested that COX inhibitors such as indomethacin (non-selective) may augment platinum-based chemotherapy in gynecologic malignancies, the addition of COX-2 inhibition to first-line therapy has been disappointing, [45] possibly due to compensatory upregulation of COX-1 and abolition of the protective effects of EP1 [46]. Long-term COX-2 inhibition produces unwanted cardiovascular events [47]. EP4 receptor inhibition may thus emerge as a bona fide treatment that circumvents the pitfalls of global COX inhibition. 

We initially explored the TCGA which provided 54 cases that were analyzed for the expression of *TUBB3*, *PTGER4*, and published biomarkers associated with clinical prognosis, *LMOD1* and *ARL4C*. *LMOD1*, leiomodin-1, is a smooth muscle cell-restricted gene that is preferentially expressed in differentiated smooth muscle cells and correlates with good outcome in extrauterine LMS. *ARL4C*, ADP ribosylation factor-like 4C, a novel Wnt target molecule, correlates with poor LMS prognosis in uterine and extrauterine LMS. Mean gene expression analysis of *LMOD1*, *ARL4C*, *TUBB3*, and *PTGER4* confirmed that *LMOD1* and *ARL4C* are overexpressed in conventional and poorly differentiated LMS, respectively; furthermore, consistent with our hypothesis, *TUBB3* expression mimicked *ARL4C*, exhibiting higher expression in the LMS subtype associated with worse prognosis. Interestingly, *PTGER4* has increased expression in both subtypes of LMS which suggests that inflammation could play an important role in both conventional and poorly differentiated LMS subtypes.

Next, we examined the expression of class III β-tubulin and EP4 in 29 cases representing a range of uterine stromal tumors. In our cohort, LMS were found to differentially express class III β-tubulin, with half exhibiting strong (2+) immunohistochemistry staining and half exhibiting no detectable staining. In contrast, only 12.5% of cases of STUMP/leiomyomatosis exhibited 2+ expression. Consistent with clinical behavior, benign leiomyomas and normal myometrial controls did not express detectable class III β-tubulin. Under-representation of early stage disease in the LMS cohort limited our ability to investigate the relationship between stage and class III β-tubulin. Our data are consistent, however, with recently published data demonstrating increased expression of class III β-tubulin in soft tissue LMS [11]. In the present study, class III β-tubulin was not predictive of overall survival, perhaps due to the limited number of cases examined. Interestingly, a phase II study of ixabepilone, a microtubule-stabilizing agent which putatively overcomes taxane resistance conferred by upregulation of class III β-tubulin, showed no activity as a second-line agent in this disease [19], underscoring gaps in our current understanding of the biology of this disease and the persistent need for novel approaches to therapy.

Consistent with our hypotheses and the role of inflammatory pathways in the setting of necrosis, total EP4 expression was higher in LMS compared to the other smooth muscle tumors that were investigated. All LMS (100%) exhibited EP4 expression ≥1+; in contrast, negative staining for EP4 was observed in 12.5% of leiomyomatosis/STUMP, 18% of leiomyomas, and 17% of normal myometrium. These findings are consistent with the literature that EP4 is upregulated in colorectal cancer [48] relative to normal colonic epithelium, malignant versus benign kidney cells [49], breast cancer [22], and castration-resistant hormone-naïve prostate cancer [50].

EP4 and other G-protein-coupled receptors are classically localized to the plasma membrane, however, the significance of nuclear localization of EP4 has increasingly been described [46,51,52]. In non-small cell lung cancer, low nuclear expression of EP4 predicts improved survival [53]; in breast cancer [22], cytoplasmic expression of EP4 more closely correlates with decreased survival in a pre-clinical model. Interestingly, bladder cancers demonstrate lower expression of cytoplasmic and nuclear EP1-4 relative to normal urothelium [54]. In the present study of LMS, low (<1+) cytoplasmic EP4 staining was associated with improved crude overall survival. Overall, our findings suggest that cytoplasmic EP4 could serve as a biomarker for aggressive LMS. This is of immense clinical importance, as the benefit of adjuvant chemotherapy for early-stage LMS following surgery has yet to be proven, and remains the subject of ongoing clinical trials (NCT01533207) [55]. Presumably, refinement of biomarkers such as class III β-tubulin and EP4 indicative of aggressive disease may identify patients most likely to benefit from therapy.

Our data suggest that EP4 may be a rational therapeutic target for uterine LMS. As monotherapy, we achieved reductions in migration of 90% and in proliferation by 7%–10% in response to small molecular weight EP4 antagonists. Dose–response effects were observed for AH23848, but not RQ15986, for unclear reasons. Importantly, pre-treatment of cells with either antagonist could successfully double sensitivity of both sarcoma and carcinoma cell lines to docetaxel. EP4 receptor antagonists have established safety in two clinical trials, and efficacy for oncologic indications are underway [53]. Treatment with EP2 or EP4 antagonists or celecoxib resulted in an increase in sensitivity of bladder cancer cells to cisplatin treatment [34]. In addition, Lin et al. demonstrated in pre-clinical models that treatment with EP4 antagonist GW627368X reduces tumor chemoresistance of breast cancer and colorectal tumors in vivo when used in combination with paclitaxel, thus demonstrating that EP4 antagonists could be a viable addition to chemotherapeutic treatment for a variety of cancers including LMS [56]. As reported in other malignancies, EP4 antagonists as single agents have modest or negligible effects on LMS cell proliferation [25,34]. The potentially interesting finding is that, in spite of this, EP4 antagonists sensitize cells to docetaxel and other chemotherapeutics.

In summary, to our knowledge this is the first report to describe class III β-tubulin expression in uterine LMS, and the first description of EP4 expression in gynecologic malignancy. Our data suggest that class III β-tubulin and EP4 expression in LMS may be used to identify patients at risk of resistance to standard chemotherapies who are candidates for augmentation of cytotoxic therapy through EP4 inhibition. EP4 antagonists are a promising adjunct to current therapy, with an acceptable side effect profile based on human studies performed by others to date. Larger studies and in vivo models are warranted.

## Figures and Tables

**Table 1:** TCGA Gene Expression Analysis.

Gene		Poorly Differentiated LMS Subtype I: *n* = 22	Conventional LMS Subtype II: *n* = 32	Tests *, *p*-Value
LMOD1				0.0002 **
	Median	3.6	6.5	
	Range	8.3	10.4	
	Mean	3.3	5.9	
	SD	2.4	2.2	
ARL4C				0.01 **
	Median	−0.003	−0.7	
	Range	6.1	5.8	
	Mean	0.4	−0.6	
	SD	1.6	1.3	
TUBB3 (β-3 Tubulin)				0.0001 **
	Median	1	−0.6	
	Range	4.7	4.3	
	Mean	0.9	−0.6	
	SD	1.4	1.2	
PTGER4 (EP4)				0.14
	Median	1.7	2.8	
	Range	5.6	6.6	
	Mean	1.5	2.2	
	SD	1.3	1.9	

LMOD1, leiomodin-1; *ARL4C,* ADP ribosylation factor-like 4C; General Linear Model F_1,52_ = 16.67, *p* = 0.0002; * Comparison of distribution of biomarkers between Subtype I and Subtype II; ** *p* ≤ 0.01.

**Table 2:** Patient Clinical Characteristics.

Average Age (Years ± SD)	All	52.18 ± 13.69	
	Leiomyosarcoma	59.2 ± 13.46	
		*n*	%
Race	White	12	41.4
	Black	16	55.2
	Hispanic	1	3.4
Smooth Muscle Type (*n* = 29)	Leiomyoma	11	37.9
	Leiomyomatosis	3	10.3
	STUMP	5	17.2
	Leiomyosarcoma	10	34.5
Leiomyosarcoma (*n* = 10)	**Stage**		
	IA	1	10
	IIIA	1	10
	IIIC	2	20
	IVB	6	60
	**Surgery**		
	TAH/BSO ± LND	6	60
	Other	4	40
	Residual Disease	2	20
	Adjuvant Therapy	6	60
	Dead of Disease	4	40

TAH = total abdominal hysterectomy, BSO = bilateral salpingo-oophorectomy; LND = lymph node dissection; STUMP = smooth muscle tumor of undetermined malignant potential.

## Supplementary Materials

Supplementary File 1
