# Supplementary figures and images for: EP4 and Class III β-Tubulin Expression in Uterine Smooth Muscle Tumors: Implications for Prognosis and Treatment
Source: Cancers (Basel). 2019 Oct 18;11(10):1590. doi: 10.3390/cancers11101590 (PMC6826612; doi:10.3390/cancers11101590)

# Class III $\beta$ Tubulin Western Blots

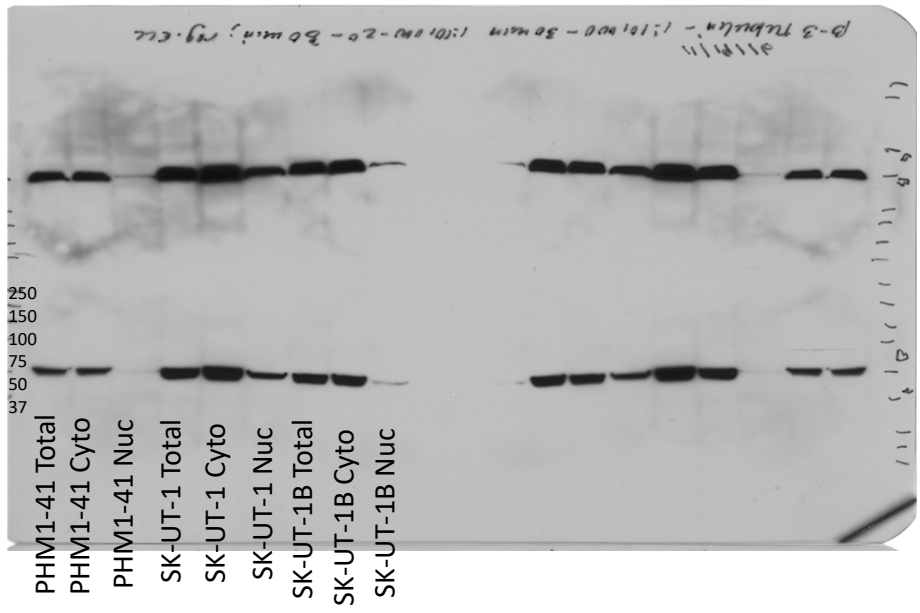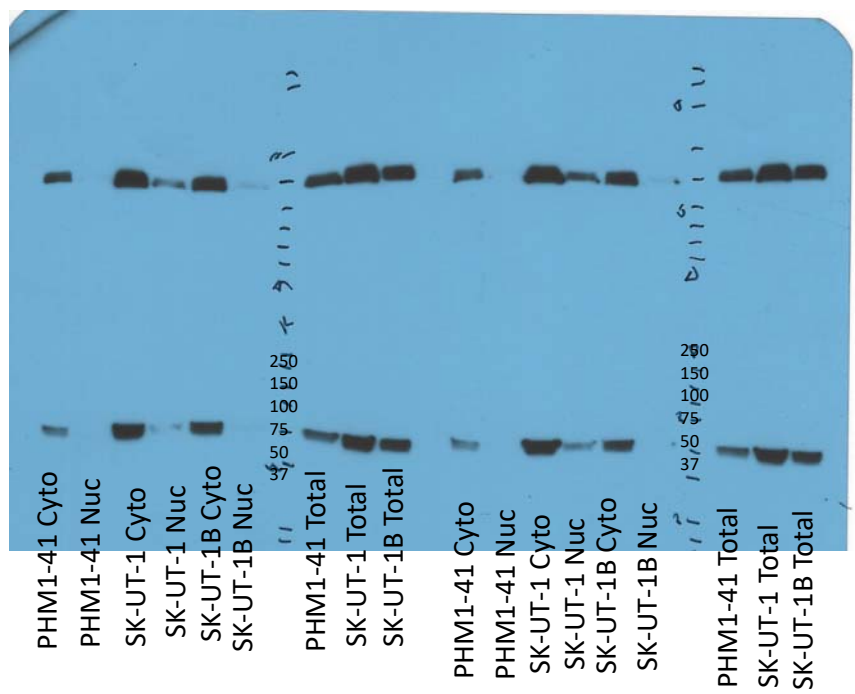

# EP4 Western Blots

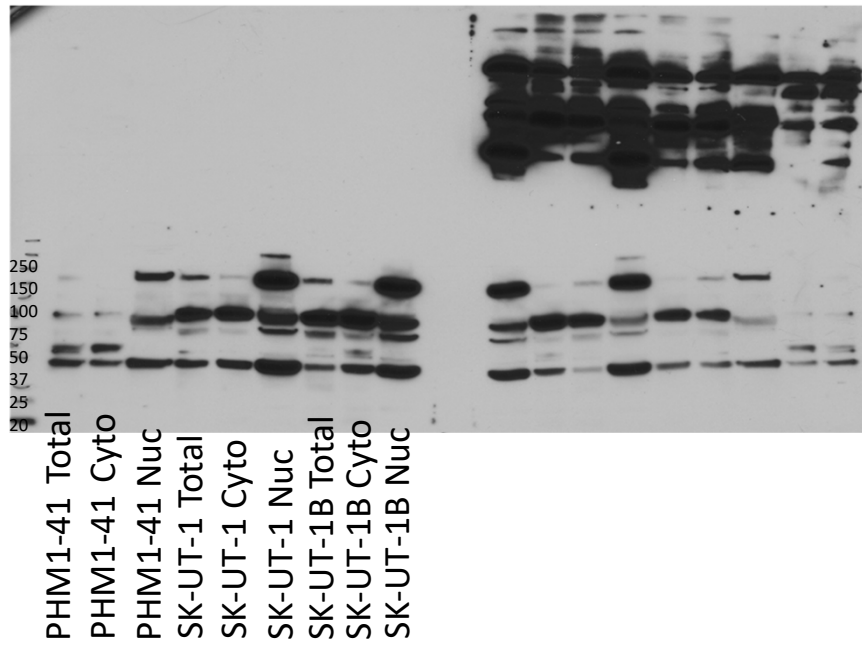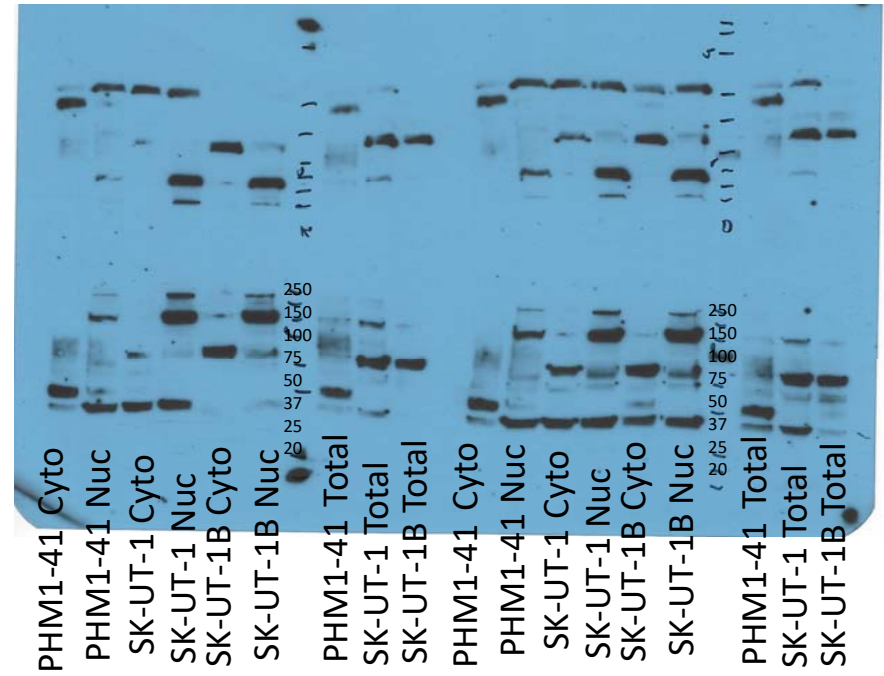

# Loading Controls

GAPDH

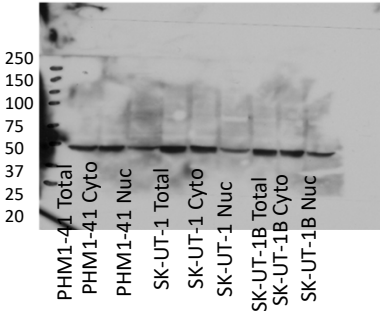

GAPDH

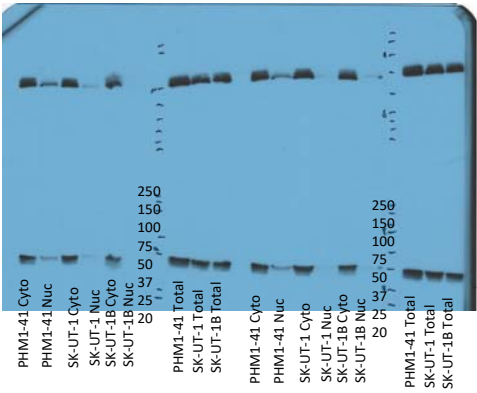

HDAC1

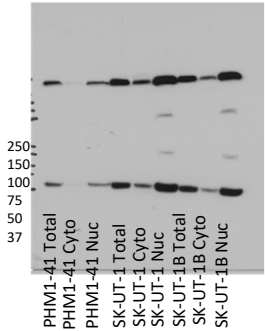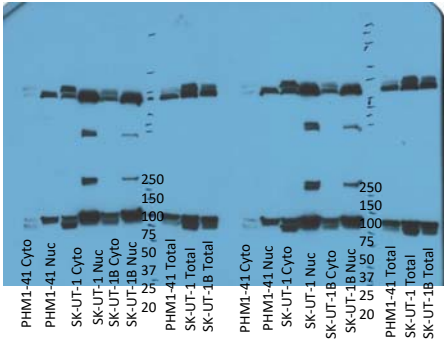

LDH

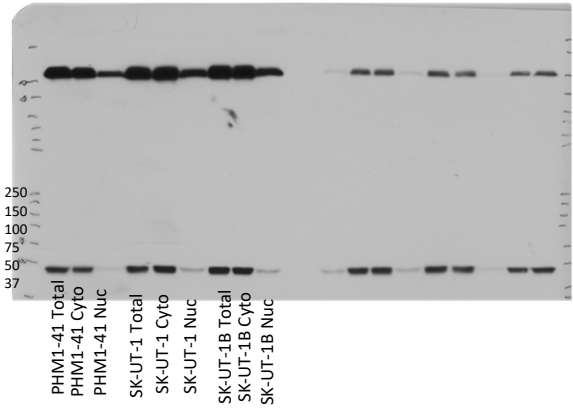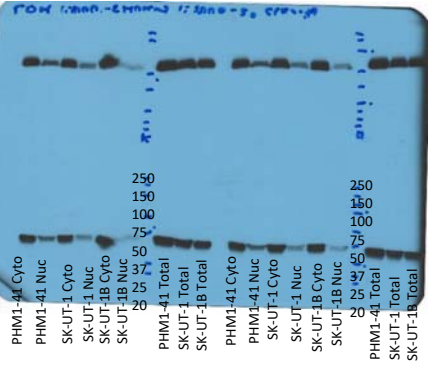

Supplement: Supplementary File 1 [file cancers-11-01590-s001.pdf]
